# Clinical safety and efficacy of microwave ablation for small renal masses

**DOI:** 10.1590/S1677-5538.IBJU.2024.0017

**Published:** 2024-04-10

**Authors:** Ashley Foret, Christopher B. Haaga, Shivani Jain, Chelsea O. Baumgartner, Megan Escott, Benjamin R. Henderson, Sean T. O'Brien, Scott E. Delacroix, Jessie R.R. Gills, Mary E. Westerman

**Affiliations:** 1 LSU Health Science Center New Orleans School of Medicine New Orleans Louisiana USA School of Medicine, LSU Health Science Center New Orleans, New Orleans, Louisiana, USA;; 2 LSU Health Science Center Department of Urology New Orleans Louisiana USA Department of Urology, LSU Health Science Center, New Orleans, Louisiana, USA;; 3 Wake Forest Department of Urology Winston-Salem NC USA Department of Urology, Wake Forest, Winston-Salem, NC, USA;; 4 East Jefferson General Hospital Department of Radiology Metairie Louisiana USA Department of Radiology, East Jefferson General Hospital, Metairie, Louisiana, USA;; 5 LSU - LCMC Cancer Center New Orleans Louisiana USA LSU - LCMC Cancer Center, New Orleans, Louisiana, USA

**Keywords:** Kidney Neoplasms, Transurethral Resection of Prostate, Radiofrequency Ablation

## Abstract

**Purpose:**

CT-guided MWA is a safe and effective tool that should be utilized in the treatment of small renal masses (SRMs). We aim to clarify the utility of CT-guided MWA by examining patient outcomes such as recurrence, treatment success, changes in renal function, and complications.

**Methods:**

A retrospective review of consecutive patients with SRMs who underwent same day renal mass biopsy (RMB) and CT-guided MWA between 2015 and 2022 was performed. Treatment safety was assessed by 30-day complications according to the Clavien-Dindo system and change in eGFR >30 days post-procedure. Treatment efficacy was defined by local recurrence and incomplete treatment rates and calculated using the Kaplan-Meier method.

**Results:**

A total of 108 renal masses were found in 104 patients. The overall complication rate was 7.4% (8/108), of which 4 were major complications (3.7%). For those with renal function available >30 days post ablation, the median eGFR was 47.2 (IQR: 36.0, 57), compared to 52.3 (IQR: 43.7, 61.5) pre-ablation, p<0.0001. 5-year local recurrence free survival was 86%. Among those with biopsy proven malignancy (n= 66), there were five local recurrences (7.54%) occurring at a median of 25.1 months (IQR 19.9, 36.2) and one case (1.5%) of incomplete treatment.

**Conclusions:**

As the medical field continues to evolve towards less invasive interventions, MWA offers a valuable tool in the management of renal masses. With low major complication and recurrence rates, our findings support the utility of CT-guided MWA as a tool for treatment of SRMs.

## INTRODUCTION

Renal cancer ranks among the top ten most prevalent malignancies worldwide, with an incidence projected to reach 81,800 cases in the United States alone in 2023. The rise in renal cancer over the past few decades can be attributed to increased detection of small renal masses (SRM). This has been facilitated by the widespread use of abdominal imaging across medical disciplines (1, 2). Presently, accepted approaches for managing renal masses encompass active surveillance, image-guided ablation, or surgical extirpation (partial or radical nephrectomy). Given that a considerable portion of incidentally detected masses are SRMs (<4 cm) and occurring predominantly in an aging population, the development of less invasive strategies is of paramount importance. Among emerging minimally invasive techniques, thermal ablation has become a focal point of attention due to its potential as a promising modality for managing SRMs.

Radiofrequency ablation (RFA) and cryotherapy have been established as standard ablative treatment modalities for renal masses and have been utilized for a longer duration compared to microwave ablation (MWA). Consequently, the existing body of research primarily concentrates on the classic ablative methods, with relatively limited focus on MWA. However, the available evidence concerning the use of MWA in the treatment of renal masses indicates predominantly positive outcomes. These outcomes include low complication and recurrence rates, comparable to other modalities (1, 3–6). MWA offers several advantages compared to RFA and cryotherapy for the treatment of SRMs. One of the major benefits of MWA is its ability to deliver higher temperatures to the target tissue, resulting in faster and more efficient ablation (7). This leads to shorter procedure times and potentially improved treatment outcomes. Additionally, MWA has been shown to have a lower risk of thermal injury to surrounding structures due to its ability to create a more precise and predictable ablation zone (7). Current data indicating alteration in renal function is similar between RFA, cryoablation, and MWA (1). These benefits position MWA as a promising alternative for the treatment of SRMs.

The growing prevalence of renal masses necessitates the implementation of treatment modalities that are both reliable and efficient. We hypothesize that MWA is a safe and effective tool that can be utilized in the treatment of SRMs. Our research consequently aims to clarify the utility of CT-guided MWA in the treatment of SRMs by examining patient outcomes such as recurrence, treatment success, changes in renal function, and complications.

## MATERIALS AND METHODS

### Patients and Data Collection

A retrospective review of electronic medical records was conducted to examine patients who underwent renal mass biopsy (RMB) at East Jefferson General Hospital between 2015 and 2022 (LSU HSC IRB #2064). Of the 265 renal mass biopsies, 108 concomitant CT-guided MWAs were performed. Three patients who received biopsies and underwent RFA were excluded. Patient demographics including age, sex, race, and ethnicity, along with clinical characteristics and baseline laboratory values, were recorded. Glomerular filtration rate (eGFR) was estimated using the CKD-Epi formula. Following renal mass detection, the size and laterality of the index lesion was documented. Renal nephrometry score data was not able to be collected as it was not consistently documented in the physician notes. Additionally, due to the time frame of the study, imaging was not available for re-review for all patients. The presence of any contralateral lesions was recorded. Notably, patients with bilateral renal masses or a history of renal cell carcinoma were not excluded from this study.

For patient convenience and to limit the need for multiple procedures/anesthesia events, our practice is to perform RMB immediately prior to the MWA procedure. This allows for a single anesthesia event and a single renal access event. All pathology was assessed in accordance with the WHO 2016 guidelines (8). Details of the biopsy and ablation procedures, including any complications, were extracted from the medical record. 30-day complications were classified according to the Clavien-Dindo system with Grade III considered major complications (9).

### CT-Guided Biopsy and MWA Procedure

RMB and MWA for all patients were performed by an experienced interventional radiologist (STB or BRH) using CT for procedure imaging guidance. Patients received either general anesthesia or monitored anesthesia care at the discretion of the anesthesiologist. Multiple cores of tissue were obtained for each biopsy to increase the accuracy of diagnosis. A pathologist was present during the procedure to confirm adequate tissue sampling. For the ablation procedure, the Neuwave LK or PR Probe (Ethicon, Rariton, New Jersey) was used in either 14, 15, 17, or 19 gauge. Ablation power utilized was at the discretion of the interventional radiologist (BRH, STOB). Ablation was performed until adequate ablative zones were achieved. Hydrodissection with either normal saline or contrast was utilized in 18 patients to prevent non-target anatomy from interfering with the ablative zones. Post-procedural and delayed imaging were obtained when indicated. Pre-procedural stent placement and/or embolization were not routinely utilized.

### Follow-up

Following RMB and MWA, patients were discharged home from the recovery room. If available, creatine levels at two additional times post-procedure were recorded (at 30> but <180 days, >365 days) for calculation for eGFR. Post-procedural scans were routinely obtained at 3 months post-procedure and then yearly in accordance with AUA guidelines (10). Incomplete treatment was defined as residual tumor enhancement on the first post-ablation scan requiring re-intervention. Tumor recurrence on subsequent scans after negative interval imaging was considered local recurrence.

## Statistical Analysis

Continuous variables were summarized using medians and interquartile ranges and compared using Wilcoxon rank sum test. Categorical variables were summarized using frequency counts and percentages and compared using chi-square test. Change in eGFR post ablation was evaluated using Wilcoxon signed-rank test. Only patients who had a creatinine available more than 30 days post-procedure were included. Five-year local recurrence-free survival was calculated using Kaplan-Meier methodology. All p-values are two sided unless specified and p<0.05 is considered significant. All analysis was performed using JMP17.2.0(SAS Institute, Raleigh, NC).

## RESULTS

One hundred and four patients undergoing 108 ablation procedures met the criteria for inclusion in this study. Baseline characteristics of the patients are recorded in Table-1. The median age for patients undergoing MWA was 72.2 (IQR 66, 78) and 60 (57.7%) were male. The median BMI was 31 (IQR 26.0, 35.9) and median eGFR for the cohort was 52.3 (IQR 42.9, 61.1). At diagnosis 17 patients (16.3%) had bilateral lesions, however most index lesions (63/108, 58.3%) were right sided. The median index tumor size was 2.5 cm (IQR 1.9, 3), with a mean of 2.44 cm (std 0.82). Nearly 20% of patients had a history of renal cell carcinoma (19, 18.3%). Six (5.6%) of these patients had a prior partial nephrectomy on the index kidney and 5 (4.6%) a prior ablation. Of the 11 salvage procedures, 4 (36.36%) of these were treatment for the same index lesion.

**Table 1:** Baseline Clinical Features of 104 Patients Undergoing Microwave Ablation.

Median Patient Age years (IQR)			72.3 (66.7, 78)
Mean Patient Age years (std)			71.34 (10.96)
Male (%)			60 (57.7%)
Caucasian (%)			85 (81.7%)
Median ECOG status (IQR)			0 (0,1)
Mean ECOG status (std)			0.56 (0.9)
Median BMI *kg/m*^2^ (IQR)			31 (26.0, 35.9)
Mean BMI *kg/m*^2^ (std)			31.4 (7.4)
Median Baseline Creatinine in *ng/dl* (IQR) (n=101)			1.0 (0.8, 1.21)
Mean Baseline Creatinine in *ng/dl* (std) (n=101)			1.1 (0.36)
Median Baseline eGFR in mg/ml (CKD-Epi) (IQR)			52.3 (42.9, 61.1)
Mean Baseline eGFR in mg/ml (CKD-Epi) (std)			55.4 (37.7)
**Tobacco Use**			
	Yes		57 (52.8%)
	No		44 (40.7%)
	Unknown		7 (6.5%)
History of Renal Cell Carcinoma, n (%)			19 (18.3%)
History of non-renal malignancy			41 (39.4%)
**Laterality (index lesion)**			
	Left		45 (41.7%)
	Right		63 (58.3%)
Solitary Kidney (%)			7 (6.5%)
Bilateral Tumors			17 (16.3%)
Prior ablation			5 (4.6%)
Prior partial nephrectomy (same side)			6 (5.6%)
Median Tumor Size in cm (IQR)			2.5 (1.9, 3)
Mean Tumor Size in cm (std)			2.44 (0.82)
**Biopsy Histology**			
	Clear Cell RCC		46 (42.6%)
		Papillary RCC	16 (14.8%)
		Papillary Type 1	11
		Papillary Type 2	3
		Papillary NOS	2
Benign			16 (14.8%)
Oncocytoma			21(19.4%)
Non-Diagnostic			2 (1.9%)
Suspicious			2 (1.9%)
Oncocytic Neoplasm (including chromophobe)			3 (2/7%)
Other			2 (1.9%)

**Figure 1:**
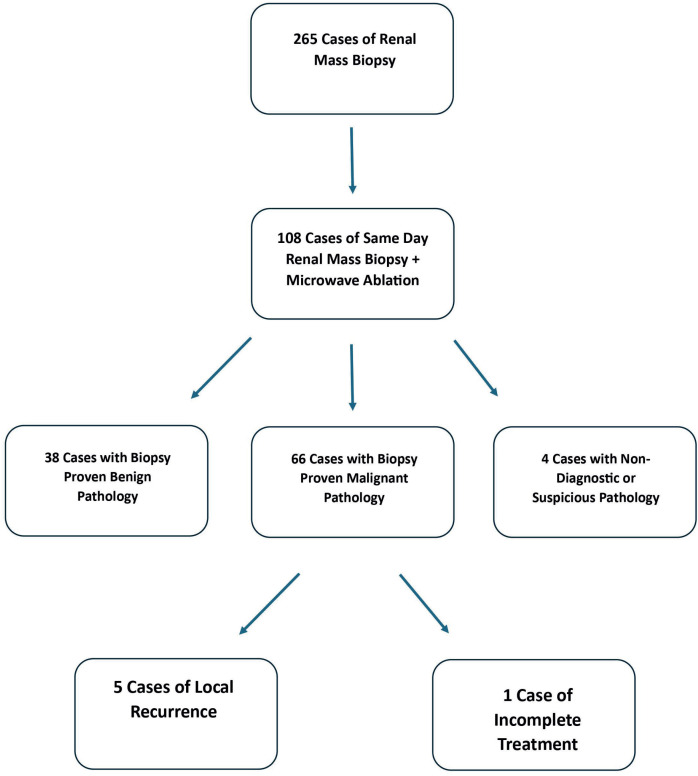
Flowchart Detailing Patient Sample.

On biopsy, 38 (20.135.1%) tumors were identified as benign, either categorized as oncocytoma (21/108, 19.4%), benign parenchyma (16/108, 14.8%) or AML (1/108, 0.9%). Four (3.7%) were non diagnostic or suspicious, while the rest (66/108, 61.1%) were malignant (Figure-1). Clear cell renal cell carcinoma was the most common malignant pathology (46/108, 42.6%). Biopsies showing benign parenchyma came from smaller masses than those showing neoplasm (including oncocytomas), although this did not reach statistical significance- median 1.9 cm (IQR 1.35, 2.85) versus 2.5 cm (IQR 2,3) p=0.08.

Table-2 shows the procedural related details. The 17-gauge probe was the most utilized, typically with a median microwave generator power of 65 watts (IQR 65, 90). The range of ablation time was 1 to 20 minutes, with a median time of 10 minutes (IQR 7,10). In total, hydro dissection was utilized in 18 procedures (16.6%) and 9 procedures (8.3%) required the use of two probes.

**Table 2:** Procedure Details for 104 Patients Undergoing Microwave Ablation.

Anesthesia Used		
	General	89 (82.4%)
	MAC	6 (5.5%)
	Unknown	13 (7.1%)
**Median Ablation Power, Watts (IQR)**		**65 (65, 90)**
	65	69 (70%)
	90	5 (5%)
	95	1 (1%)
	100	19 (19%)
	140	3 (3%)
	Multiple	2 (2%)
Mean Ablation Power, Watts (STD)		75.16 (19.8)
Median Ablation Time, minutes (IQR)		10 (7,10)
Mean Ablation Time, minutes (STD)		9.1 (3.8)
**Probe Size, gauge (%)**		
	14	18 (16.7%)
	15	3 (2.8%)
	17	75 (69.4%)
	19	1 (0.9%)
	n/a	2 (1.9%)
Number of Ablations Requiring 2 Probes		9 (8.3%)
**Hydrodissection**		**18 (16.7%)**
	Normal Saline	10
	Contrast	8
Immediate post-procedure admission		9 (8.3%)
Median Length of stay for those requiring admission, days (IQR)		1 (1,1)
Delayed Imaging		74 (68.5%)

Post procedure, there were eight admissions (7.4%) for observation, with a median length of stay of 1 day (IQR 0,1) for admitted patients. Two patients (1.9%) required rehospitalization following procedure discharge, one for a duodenal perforation and one for a pleural effusion the rest were discharged home the same day. There were 8 (7.4%) total complications, four (3.70%) of which were major, detailed in Table-3. In total 66 patients had post-procedural creatinine available more than 30 days following the ablation. For this cohort, the median pre-procedural eGFR was 52.3 (IQR: 43.7, 61.5) and median eGFR >30 days post-procedural was 47.2 (IQR: 36.0, 57), p<0.0001.

**Table 3:** 30-day procedural related complications for 108 patients undergoing renal mass biopsy and microwave ablation.

Clavien Grade	n	Description	Treatment
I	1	Urinary retention	Catheter placement
	2	Renal hematoma	No intervention required; post-operative monitoring via CT-Imaging
	1	CHF exacerbation.	Overnight stay for monitoring
IIIa	1	Intraoperative pleural effusion	Thoracentesis requiring local anesthesia
IIIb	1	Duodenal Perforation	CT-guided drain placement by Interventional Radiology
	1	Pneumothorax	Treated intra-op. Resorption of pneumothorax using a 5F pigtail catheter
	1	Collecting System Extravasation	Perinephric drain placement by Interventional Radiology

**Figure 2:**
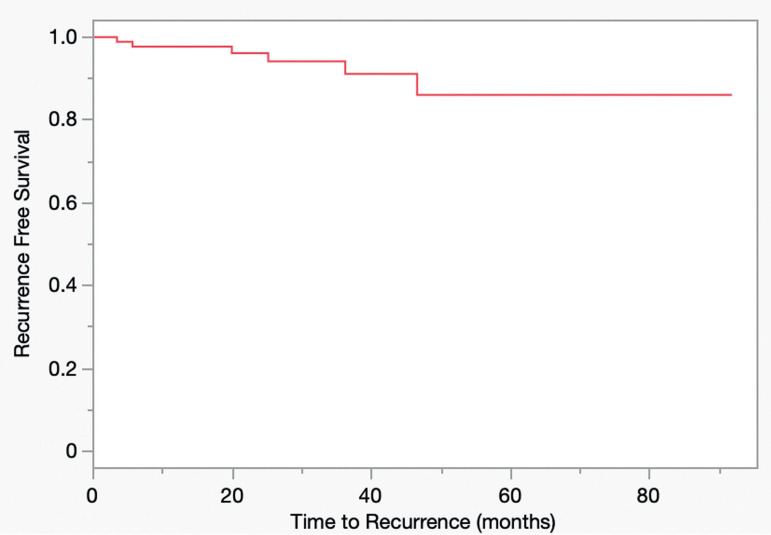
Kaplan-Meier Curve Showing Time to Local Recurrence following Renal Mass Ablation.

The median duration of follow up for those alive at last follow up was 22.68 months (IQR: 3.3, 26.6). During this time, a total of four patients died, one of whom died with metastatic renal cell carcinoma. This patient underwent a nephrectomy and lobectomy 4 years prior to contralateral mass ablation. Five years recurrence free survival was 86% (Figure-2). A total of six patients with biopsy proven malignant pathology required further treatment in the same kidney for renal cell carcinoma. Among those with biopsy proven malignancy (n= 66), there were five local recurrences (7.54%) occurring at a median of 25.1 months (IQR 19.9, 36.2) post ablation and 1 case of incomplete treatment identified on the 3-month post-ablation CT scan. Of the 5 recurrences, 3 occurred in the same focus as the ablated neoplasm and 2 were in novel foci. Among the entire cohort, there were 0 malignant recurrences in patients with biopsy-proven oncocytoma or benign parenchyma.

Among those requiring additional treatment, management techniques varied. In the case of incomplete treatment, the patient underwent nephrectomy with final pathology showing clear cell renal cell carcinoma. Two patients with local recurrence received repeat ablation and three underwent active surveillance.

## DISCUSSION

Our findings endorse the application of MWA as a therapeutic modality for managing SRMs. Accompanied by a brief procedural duration, MWA demonstrated low incidence of complications, incomplete treatment, and recurrence. Consequently, our dataset reinforces the viability of MWA for the treatment of SRMs (<4cm). This endorsement is particularly noteworthy for individuals within the aging demographic, as our median patient age was 72. Additional benefits in treatment with MWA could be provided for those navigating intricate comorbidities that contraindicate prolonged anesthesia.

This data demonstrated major complication rates, incomplete treatment rates, recurrence rates and procedure times similar to previous research performed on MWA (11–14). Overall, our recurrence rates appear to be slightly higher than other available literature examining MWA. There are several possible reasons for this difference. Our study differs from other pieces of literature in the sample size and study duration. The sample size of renal masses treated with CT-guided MWA is nearly double that of other available studies examining this modality. Most literature that exists was published in a shorter time such as two years, unlike our ability to examine recurrence in patients treated as early as 2015. In comparison to other modalities of treatment, our local recurrence rate of 7.04% is similar to those demonstrated in other studies. Additionally, our major complication rate of 3.70% appears to be consistent or lower than treatment with other modalities (1, 15, 16). Systemic reviews have been made available which directly compare the results of the different treatment modalities. In a metanalysis comparing MWA and cryoablation for treatment of SRMs, MWA showed improved 1-year local tumor recurrence in addition to lower ablation durations. Additional research comparing all three treatment modalities supports similar functional or surgical outcomes between the ablation methods, with lower complication rates for MWA (17). This is consistent with our results. Our study also reveals a median procedure time of just 10 minutes for MWA, highlighting its efficiency in comparison to cryoablation and RFA. This aligns with existing research indicating that patients undergoing MWA require less time under anesthesia (17). Despite these promising findings, it's important to note that current guidelines do not include recommendations for MWA utilization. However, our work contributes valuable evidence supporting the integration of MWA into clinical practice. By showcasing its safety profile and shorter procedure duration when contrasted with cryoablation and RFA, we believe that wider adoption of MWA could be warranted. Further investigation incorporating our findings is needed to comprehensively compare MWA with other less invasive treatment modalities.

MWA has been determined to be a safe treatment modality with respect to renal function (18, 19). Our data revealed that there was a 5-point decrease in eGFR > 30 days post-procedure in comparison to pre-procedure eGFR. This minimal decrease corroborates other research examining the effects on renal function >6 months post MWA (19). Prior studies indicate that the largest decrease in renal function following MWA will occurs <30 days post procedure (18). By examining GFR from >30 days post MWA, we can infer the long-term, likely insignificant effects on renal function. In comparison to other treatment modalities, available research supports the usage of MWA. A study by Zhou et al. showed no difference in immediate changes in renal function between the ablative modalities (1) When examining MWA and laparoscopic radical nephrectomy, renal function is substantially less damaged following treatment with MWA (20). Our research affirms MWA as a treatment for SRM with the added benefit of preserved renal function.

RMB rarely precedes surgical therapy, despite prior studies reporting that around 20% of cT1 lesions reveal benign findings on post-surgical pathology reports (21). In line with prior literature, approximately 20% of patients who underwent concurrent ablation at time of biopsy ultimately had benign pathology, overwhelmingly oncocytoma. Concomitant biopsy and ablation leading to overtreatment of benign lesions has been reported in other studies as well. Additionally, research has shown a greater chance of histological diagnoses in comparison to non-diagnostic pathology when biopsy and ablation are separated (22). The use of staged renal biopsy and ablation has been significantly increasing since 2012. Although there is a greater expense associated with separate interventions, staged biopsy and ablation does not only limit the overtreatment of benign masses but also has less chance of inpatient stay and readmissions post-operatively (23). Currently, these benefits must be evaluated against the additional costs. Future efforts to limit costs of the staged method should be considered.

While the study presents evidence in favor of MWA in the treatment for SRMs, it is essential to acknowledge the study's limitations. First, the study's population is overwhelmingly Caucasian which may potentially impact the generalizability of the results. Though the follow-up period is longer than many other studies of its kind, we currently do not have long-term data of 10+ years, precluding a completely comprehensive understanding of MWA's durability and potential recurrence rates. Additionally, the experience of the interventional radiologist conducting the MWA procedure has the potential to influence the rates of incomplete treatment and complications. To address these limitations, future prospective studies with larger and more diverse cohorts, extended follow-up periods, and comparative analyses against alternative treatment methods would be invaluable in further elucidating the true potential and limitations of MWA.

Additionally, because nephrometry score was not consistently documented in physician notes and due to the time frame of the study, imaging was not available for re-review for all patients, we were not able to include it in our analysis. Future studies evaluating the association between nephrometry score and microwave ablation outcomes could be beneficial. Other enhanced decision-making processes for the conservative management of complex renal masses may include utilizing preoperative 3D models to develop a tailored strategy according to patient and tumor characteristics (24). In addition, identification of patients at risk for pathologic upstaging could improve treatment efficacy. Utilization of models such as those previously described by Cao et al, which incorporates tumor maximum and minimum diameter, fibrinogen, and tumor size could also aid in better patient selection for ablative procedures (25).

## CONCLUSION

This research makes a compelling case for the integration of MWA into the armamentarium of renal mass treatments. The evidence provided demonstrates not only the technical feasibility and safety of this approach, but also its potential to improve patient outcomes and quality of life. As the medical field continues to evolve towards less invasive interventions, MWA offers a valuable tool in the management of renal masses. Nonetheless, further research and long-term follow-up studies will be essential to solidify the technique's role in the broader landscape of renal mass treatment strategies.

## References

[1] Zhou W, Arellano RS (2018). Thermal Ablation of T1c Renal Cell Carcinoma: A Comparative Assessment of Technical Performance, Procedural Outcome, and Safety of Microwave Ablation, Radiofrequency Ablation, and Cryoablation. J Vasc Interv Radiol.

[2] Chow WH, Devesa SS, Warren JL, Fraumeni JF (1999). Rising incidence of renal cell cancer in the United States. JAMA.

[3] Mershon JP, Tuong MN, Schenkman NS (2020). Thermal ablation of the small renal mass: a critical analysis of current literature. Minerva Urol Nefrol [Internet].

[4] Hui GC, Tuncali K, Tatli S, Morrison PR, Silverman SG (2008). Comparison of percutaneous and surgical approaches to renal tumor ablation: metaanalysis of effectiveness and complication rates. J Vasc Interv Radiol.

[5] Filippiadis DK, Gkizas C, Chrysofos M, Siatelis A, Velonakis G, Alexopoulou E (2018). Percutaneous microwave ablation of renal cell carcinoma using a high power microwave system: focus upon safety and efficacy. Int J Hyperthermia.

[6] Martin J, Athreya S (2013). Meta-analysis of cryoablation versus microwave ablation for small renal masses: is there a difference in outcome?. Diagn Interv Radiol [Internet].

[7] Thompson SM, Schmitz JJ, Thompson RH, Weisbrod AJ, Welch BT, Viers BR (2018). Introduction of Microwave Ablation Into a Renal Ablation Practice: Valuable Lessons Learned. AJR Am J Roentgenol.

[8] Moch H, Cubilla AL, Humphrey PA, Reuter VE, Ulbright TM (2016). The 2016 WHO Classification of Tumours of the Urinary System and Male Genital Organs-Part A: Renal, Penile, and Testicular Tumours. Eur Urol.

[9] Clavien PA, Barkun J, de Oliveira ML, Vauthey JN, Dindo D, Schulick RD (2009). The Clavien-Dindo classification of surgical complications: five-year experience. Ann Surg.

[10] Campbell SC, Clark PE, Chang SS, Karam JA, Souter L, Uzzo RG (2021). Renal Mass and Localized Renal Cancer: Evaluation, Management, and Follow-Up: AUA Guideline: Part I. J Urol.

[11] Yu J, Wang H, Cheng ZG, Liu FY, Li QY, He GZ (2022). A multicenter 10-year oncologic outcome of ultrasound-guided percutaneous microwave ablation of clinical T1 renal cell carcinoma: will it stand the test of time?. Eur Radiol.

[12] Mansilla AV, Bivins EE, Contreras F, Hernandez MA, Kohler N, Pepe JW (2017). CT-Guided Microwave Ablation of 45 Renal Tumors: Analysis of Procedure Complexity Utilizing a Percutaneous Renal Ablation Complexity Scoring System. J Vasc Interv Radiol.

[13] Guan W, Bai J, Liu J, Wang S, Zhuang Q, Ye Z (2012). Microwave ablation versus partial nephrectomy for small renal tumors: intermediate-term results. J Surg Oncol.

[14] Ierardi AM, Puliti A, Angileri SA, Petrillo M, Duka E, Floridi C (2017). Microwave ablation of malignant renal tumours: intermediate-term results and usefulness of RENAL and mRENAL scores for predicting outcomes and complications. Med Oncol.

[15] Permpongkosol S, Link RE, Solomon SB, Kavoussi LR (2006). Results of computerized tomography guided percutaneous ablation of renal masses with nondiagnostic pre-ablation pathological findings. J Urol.

[16] Thompson RH, Atwell T, Schmit G, Lohse CM, Kurup AN, Weisbrod A (2015). Comparison of partial nephrectomy and percutaneous ablation for cT1 renal masses. Eur Urol.

[17] Pandolfo SD, Carbonara U, Beksac AT, Derweesh I, Celia A, Schiavina R (2023). Microwave versus cryoablation and radiofrequency ablation for small renal mass: a multicenter comparative analysis. Minerva Urol Nephrol.

[18] De Cobelli F, Papa M, Panzeri M, Colombo M, Steidler S, Ambrosi A (2020). Percutaneous Microwave Ablation Versus Cryoablation in the Treatment of T1a Renal Tumors. Cardiovasc Intervent Radiol.

[19] Lucignani G, Rizzo M, Ierardi AM, Piasentin A, De Lorenzis E, Trombetta C (2022). Percutaneous Microwave Ablation is Comparable to Cryoablation for the Treatment of T1a Renal Masses: Results From a Cross-Sectional Study. Clin Genitourin Cancer.

[20] Yu J, Zhang G, Liang P, Yu XL, Cheng ZG, Han ZY (2015). Midterm results of percutaneous microwave ablation under ultrasound guidance versus retroperitoneal laparoscopic radial nephrectomy for small renal cell carcinoma. Abdom Imaging.

[21] Patel AK, Lane BR, Chintalapati P, Fouad L, Butaney M, Budzyn J (2021). Utilization of Renal Mass Biopsy for T1 Renal Lesions across Michigan: Results from MUSIC-KIDNEY, A Statewide Quality Improvement Collaborative. Eur Urol Open Sci.

[22] Wells SA, Wong VK, Wittmann TA, Lubner MG, Best SL, Ziemlewicz TJ (2017). Renal mass biopsy and thermal ablation: should biopsy be performed before or during the ablation procedure?. Abdom Radiol (NY).

[23] Uhlig A, Lenis A, Wang X, Shuch B (2021). Sequencing of Renal Mass Biopsy and Ablation: Results from the National Cancer Database. Urol Pract.

[24] Grosso AA, Lambertini L, Di Maida F, Gallo ML, Mari A, Minervini A (2022). Three-dimensional reconstruction and intraoperative ultrasonography: Crucial tools to safely approach highly complex renal masses. Int Braz J Urol.

[25] Cao C, Kang X, Shang B, Shou J, Shi H, Jiang W (2022). A novel nomogram can predict pathological T3a upstaged from clinical T1a in localized renal cell carcinoma. Int Braz J Urol.

